# Expanding *TBCE*-related phenotype—novel variant causing rigid spine, eosinophilia, neutropenia, and nocturnal hypoxemia

**DOI:** 10.1007/s13353-024-00894-9

**Published:** 2024-08-17

**Authors:** Magdalena Badura-Stronka, Adam Sebastian Hirschfeld, Evgenia Globa, Anna Winczewska-Wiktor, Anna Potulska-Chromik, Anna Kostera-Pruszczyk, Dorota Wicher, Maciej Robert Krawczyński

**Affiliations:** 1 Chair and Department of Medical Genetics, Poznan University of Medical Sciences, Poznan, Poland; 2 Centers for Medical Genetics GENESIS, Poznan, Poland; 3 Department of Pediatric Endocrinology, Ukrainian Research Center of Endocrine Surgery, Endocrine Organs and Tissue Transplantation, Kiev, Ukraine; 4 Chair and Department of Developmental Neurology, Poznan University of Medical Sciences, Poznan, Poland; 5 Department of Neurology, Medical University of Warsaw, Warsaw, Poland; 6 Department of Medical Genetics, Children’s Memorial Health Institute, Warsaw, Poland

**Keywords:** TBCE, Rigid spine, KCS1, Kenny-Caffey syndrome

## Abstract

We report three patients with the novel variant c.100 + 1G > A of the *TBCE* gene and describe the presented clinical phenotype in detail. We also systematically reviewed the literature for clinical similarities and dissimilarities among all known patients with pathogenic *TBCE* variants. The clinical phenotype observed in patients with pathogenic *TBCE* variants is broader than previously described. Homozygous carriers of the c.100 + 1G > A variant exhibit a markedly milder clinical course, with no deviations in the calcium-phosphate metabolism and central nervous system pathology in MRI studies. Additionally, two patients manifest highly specific symptoms such as a rigid spine, eosinophilia, neutropenia, and nocturnal hypoxemia. Furthermore, cryptorchidism was observed in male patients. The identification of the pathogenic c.100 + 1G > A variant has thus far been limited to patients of Central-Eastern European descent, suggesting a potential founder mutation in this population.

**Supplementary information:**

The online version contains supplementary material available at 10.1007/s13353-024-00894-9.

## Introduction

Tubulinopathies are a group of severe neurodevelopmental disorders characterized by dysregulation of tubulin, a critical structural protein involved in the formation of microtubules, essential cytoskeleton components. Mutations in genes encoding tubulin isoforms or proteins associated with tubulin function lead to aberrant microtubule dynamics, disrupting intracellular transport, cell division, and migration (Parvari et al. 2007). Inactivating mutations within the *TBCE*, which encodes a tubulin folding cofactor E essential for tubulin folding and polymerization, have been established as etiological contributors to two rare autosomal recessive syndromes: hypoparathyroidism-retardation-dysmorphism syndrome (HRDS) and Kenny-Caffey syndrome type 1 (KCS1) (Parvari et al. 2002). HRDS manifests with congenital hypoparathyroidism, intellectual disability, facial dysmorphism, and profound growth failure. The KCS1 phenotype shares similarities with HRDS but is differentiated by osteosclerosis and recurrent bacterial infections as additional clinical features (Hershkovitz et al. 2007). Although both syndromes are still presented in the literature, their shared molecular basis concerning the *TBCE* suggests that they are part of a single genetic syndrome with variable expression depending on the location and type of mutation (Kammenga 2017). It is also noteworthy that Kenny-Caffey syndrome type 2 (KCS2), a genetic syndrome with many clinical similarities to KCS1, is often difficult to separate based solely on the symptoms spectrum (Schigt et al. 2023). KCS2 is an autosomal dominant syndrome caused by pathogenic variants in the family with sequence similarity 111 member A gene (*FAM111A*). *FAM111A* is involved in DNA replication and defense against viral infections (Kojima et al. 2020; Tarnita et al. 2018). The primary distinguishing features between KCS1 and KCS2 are suggested to include the presence of intellectual disability in KCS1 and macrocephaly, cortical thickening, and medullary stenosis of the long bones in KCS2 (Schigt et al. 2023).

The vast majority of children with HRDS/KCS1 originated from inbred families of Middle Eastern origin and carried a homozygous in-frame deletion c.155_166del; p.(Ser52_Gly55del) in *TBCE* (Parvari et al. 2002). So far, at least 43 patients with this pathogenic variant have been reported in this population (Touati et al. 2019). Two additional variants in a compound heterozygous state c.66_67del; p.(Val23fs) and c.1113 T > A; p.(Cys371Ter) have been reported in a Belgian pedigree manifesting features of HRDS (Parvari et al. 2002). Other investigators found that *TBCE*-related clinical phenotype is broader than that described regarding HRDS/KCS1 and varies widely. Such a case was made for five patients from Southern Italy with homozygous variant c.464 T > A; p.(Ile155Asn) in *TBCE* (Sferra et al. 2016). Additionally, one child with compound heterozygous variants c.464 T > A; p.(Ile155Asn) and c.1076del; p.(Leu360Ter) and a second with c.464 T > A; p.(Ile155Asn) and c.924del; p.(Leu309Ter) were reported in the same population (Sferra et al. 2016; Battini et al. 2021).

We want to expand further known *TBCE*-related phenotypes by presenting three patients from the Eastern European population with a *TBCE* variant (c.100 + 1G > A) and unique clinical manifestations. Additionally, we compare the carriers of different *TBCE* pathogenic variants described so far.

## Patients and methods

**Fig. 1:**
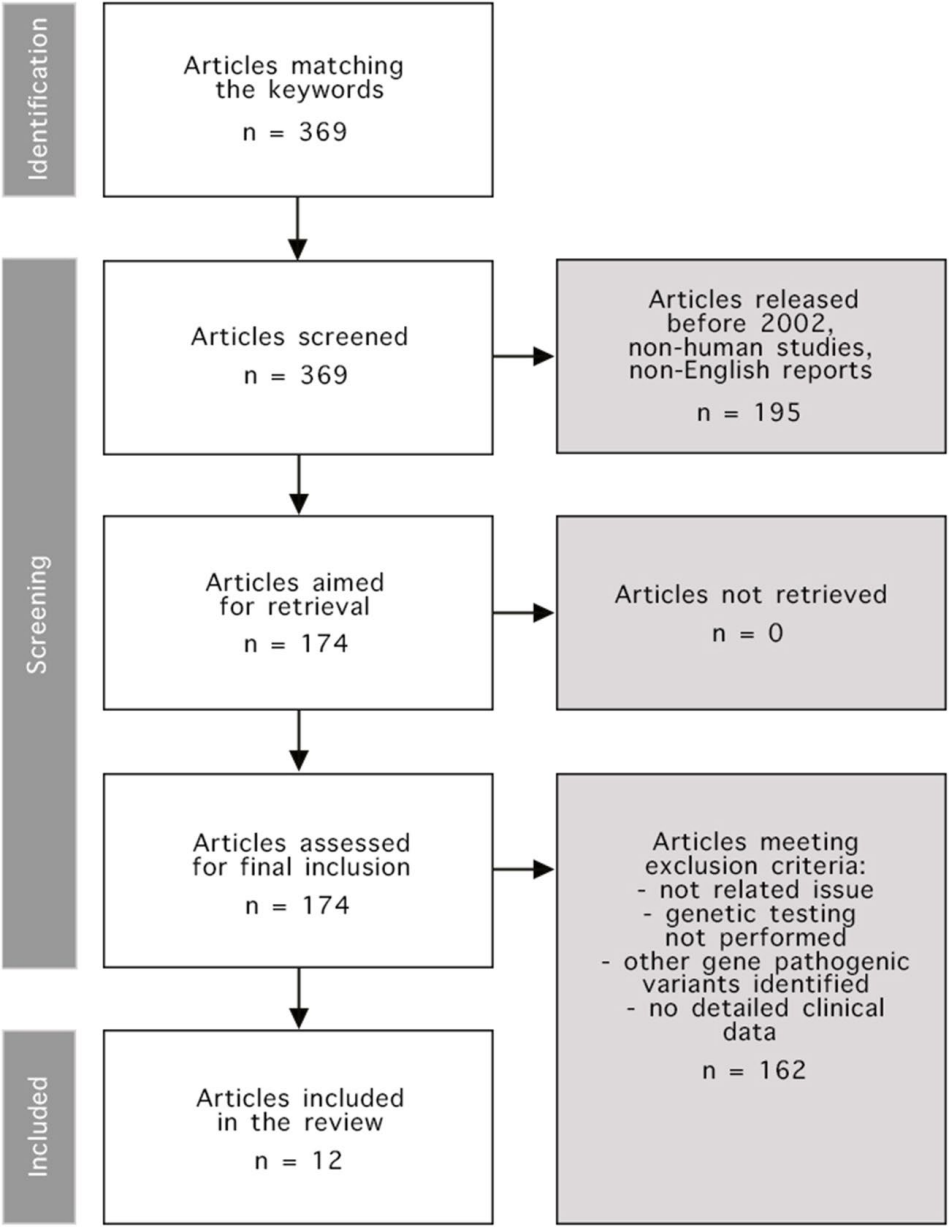
Systematic review protocol. To identify the articles we were interested in as precisely as possible, we used (((Kenny-Caffey syndrome) OR (KCS1)) OR (TBCE)) OR (HRDS) keywords in the PubMed database

Whole exome sequencing of genomic DNA was performed in two cases of Polish and one case of Ukrainian patients. Further retrospective review of the clinical characteristics and diagnostic findings was made. A detailed description of the genetic testing methods used by laboratories is provided in Supplementary Table 1. An additional systematic review of the clinical phenotype of available *TBCE*-related cases from the PubMed database search was performed per the recommendations of Preferred Reporting Items for Systematic Reviews and Meta-Analyzes (PRISMA) (Shamseer et al. 2015). Two independent reviewers conducted the review process. The review protocol is shown in Fig. 1. Due to identifying the pathogenic nature of *TBCE* variants in 2002, we started reviewing articles from that date. We only included reports with detailed descriptions of the clinical features in individual or series of cases; thus, all non-human reports were excluded. Another inclusion criterion was the definitive genetic identification of the *TBCE* variant. We only analyzed the English-language reports. Ultimately, 12 articles were included. Detailed data retrieved and analyzed from those reports is shown in Supplementary Table 2.

## Results

A homozygous pathogenic variant c.100 + 1G > A in *TBCE* was detected in three patients (P1-P3), followed by the confirmation of heterozygous carrier status of the variant in their parents. This variant has not been previously described in scientific literature in individuals exhibiting *TBCE*-related symptoms. The ClinVar database provides information about conflicting pathogenicity classifications for this variant based on current submissions from genetic laboratories: pathogenic (2), likely pathogenic (3), uncertain significance (1).

In the c.100 + 1G > A variant, the sequence alteration impacts a donor splice site within intron 2 of the *TBCE*, potentially disrupting RNA splicing. Mutations that interfere with the donor or acceptor splice site generally result in a loss of protein function (Baralle and Baralle 2005). A systematic review of available reports successfully identified 98 patients with a confirmed pathogenic variant in the *TBCE* (Parvari et al. 2002; Hershkovitz et al. 2007; Sferra et al. 2016; Battini et al. 2021; Aminzadeh et al. 2020; Moussaid et al. 2012; Padidela et al. 2009; Albaramki et al. 2012; Ratbi et al. 2015; Kerkeni et al. 2015; 

Hafez et al. 2017; Naguib et al. 2009). Within this cohort, most (91/98) presented cases were confirmed with pathogenic variants characterized by a homozygous deletion c.155_166del. In 5/98 cases, a homozygous point mutation c.464 T > A was confirmed. In 2/98 cases, a combination of a point mutation and a deletion was observed: c.464 T > A; c.1076del and c.464 T > A; c.924del.

### Clinical summary—P1

Patient 1 is a 13-year-old girl of Polish descent with no significant family history. She was born at term with a normal birth weight and did not exhibit any congenital anomalies. Her gross and fine motor skills developed appropriately, achieving independent ambulation at 14 months. However, there was a slight delay in speech development. The initial symptoms manifested at age 6, presenting as muscle weakness and limited mobility in the cervical spine. Increased muscle tone around the paraspinal muscles was noted. Over the subsequent years, the symptoms of a rigid spine progressed, involving both the thoracic and lumbar segments. Since the age of 11, an exacerbated thoracic spine scoliosis was observed. At this stage, the patient was suspected of having rigid spine muscular dystrophy 1 (RSMD) or spondyloarthropathy. A neurological examination at age 13 revealed reduced muscle strength in proximal muscles, atrophy of limb muscles, diminished tendon reflexes, and waddling gait. Body weight and height were below the 3rd percentile. Nocturnal hypoxemia was noted. The overall sleep duration was 517.6 min, during which 115 respiratory events were observed: 20 central apneas, 37 obstructive apneas, and 14 mixed apneas. The median duration of central apneas was 16.4 s, with the longest lasting 24 s; obstructive apneas had a median duration of 18.1 s, with the longest lasting 37 s. Most of these events occurred during the Non-Rapid Eye Movement (NREM) sleep phase. Nocturnal noninvasive ventilation was started. Additionally, densitometry results indicated osteoporosis (Z-score − 1.5, T-score − 3.9), and blood serum analysis revealed eosinophilia, neutropenia, and platelet anisocytosis (without thrombocytopenia). A conducted WES study (at the age of 13) identified a homozygous variant c.100 + 1G > A in the *TBCE*.

### Clinical summary—P2

**Fig. 2:**
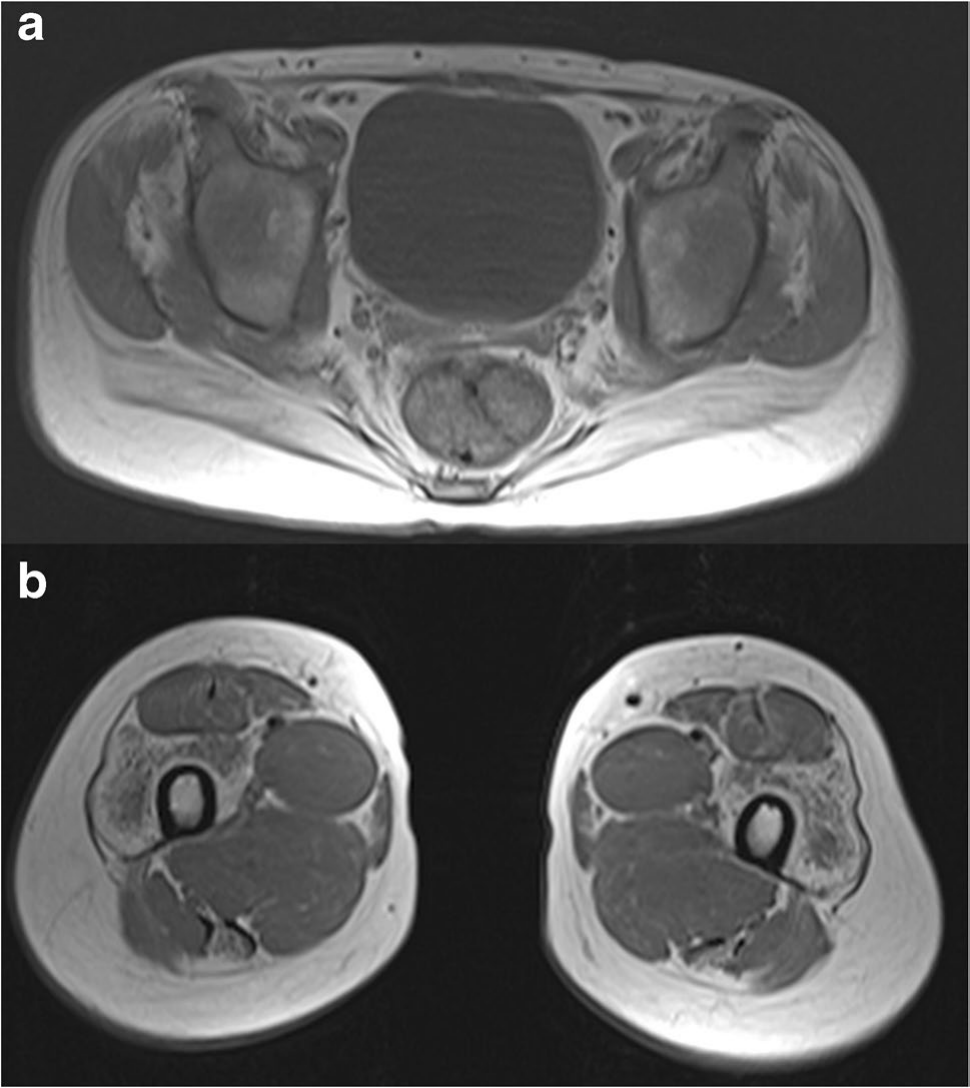
The MRI of limb-girdle muscles in patient 2 with *TBCE* c.100 + 1G > A homozygous variant shows **a** atrophy of the gluteus muscle group, mainly pronounced in gluteus maximus muscles, accompanied by fatty infiltration, **b** muscle atrophy and fatty infiltration in the vastus lateralis and semitendinosus muscles in the T1 sequence

**Fig. 3:**
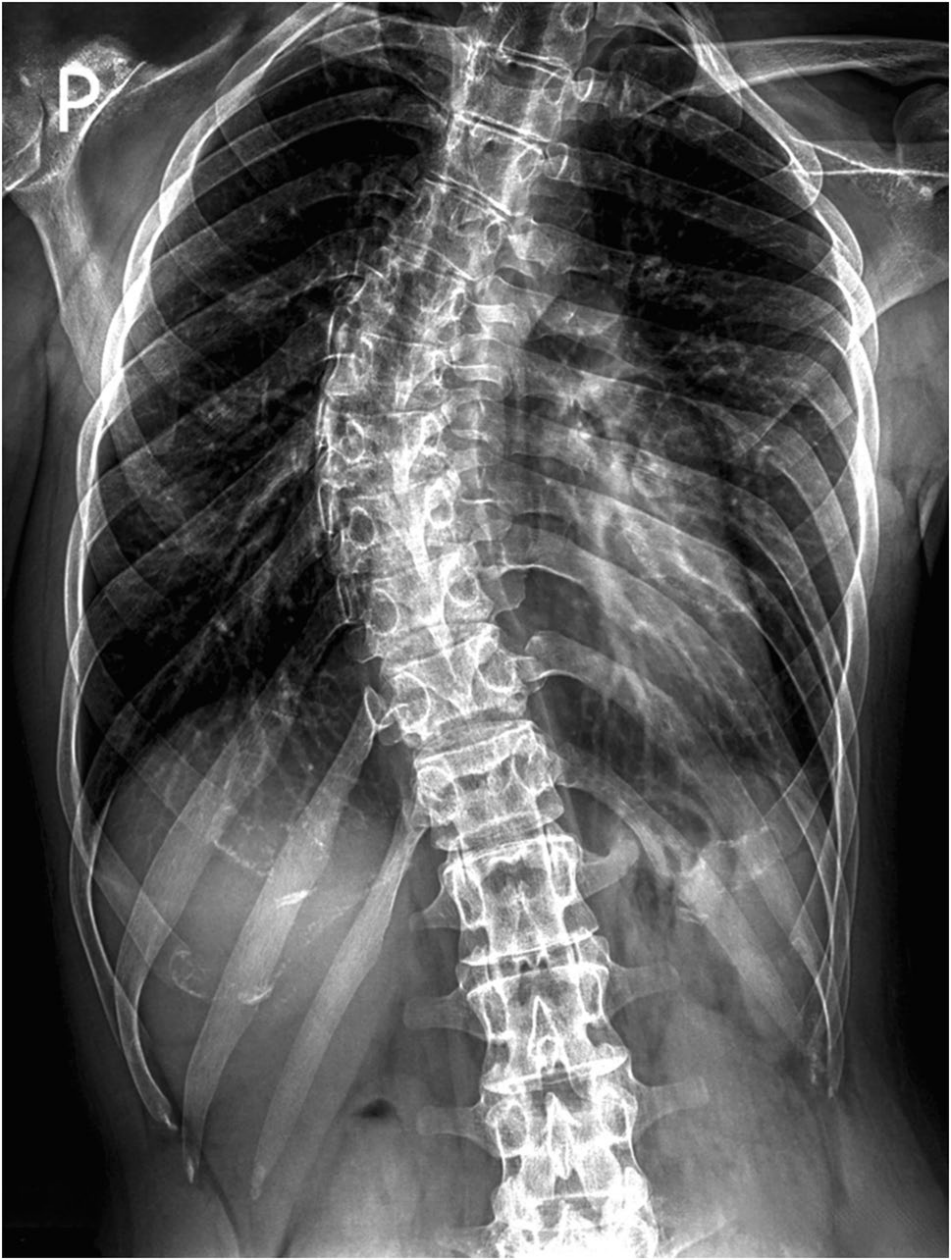
The spine x-ray shows thoracic scoliosis in patient 2 with *TBCE* c.100 + 1G > A homozygous variant

Patient 2 is a 20-year-old man of Polish descent with no significant family history. He was born at term with a normal birth weight. Bilateral cryptorchidism was noted. Motor development occurred with discrete delay, achieving independent sitting at 9 months and independent walking at 15 months. Speech development was also delayed; at age 4, he used single words. Health issues first arose at the age of 4, with febrile seizures during pneumonia. A total of 5 febrile seizure episodes occurred, with no recurrence after the age of 5. Pneumonia episodes continued to occur periodically. At the age of 5, generalized muscle pain emerged. The neurological examination at 11 noted difficulty rising from a squat with support, distal limb paresis, and contractures in the shoulder, elbow, and ankle joints. Subsequent neurological evaluation at the age of 13 revealed limited spine mobility, muscle atrophy primarily affecting the shoulder girdle, and the onset of nocturnal hypoxemia events. Nocturnal noninvasive ventilation was started. The muscular tissue was assessed using MRI while examining the thoracic and lumbar spine and the sacroiliac joints. A notable atrophy of the paraspinal muscles was observed. However, there was no evidence of atrophy or edema in the chest wall muscles, upper girdle, and upper limbs. On both sides, the iliopsoas and psoas muscles exhibited signs of significant fatty degeneration inconsistent with the patient’s age, with moderate steatosis in the buttock muscles. The cervical spine MRI showed kyphotic alignment in the C3-C5 segment, with a left-sided border curvature at the C-Th junction. Noted deformities affected the intervertebral joints in the C2-C4 region without evidence of the spinal canal or intervertebral foramina stenosis. There were no symptoms indicative of bony blocks. A partial cleft in the arches of the C2-C3 vertebrae was detected. At 14, gynecomastia and abnormal densitometry results (Z-score 0.3, T-score − 1.5) were observed. At 17, the MRI of limb-girdle muscles and lower limb muscles was performed. Significant atrophy in the paraspinal muscles of the lumbosacral section, iliopsoas, and tibialis posterior was noted. Furthermore, moderate atrophy in the gluteal muscles, vastus muscles of the thigh, semitendinosus muscles, and, to a small extent, in the rectus femoris, adductor magnus, and the lateral head of the gastrocnemius muscles was present (Fig. 2). The muscles of the abdominal wall were preserved. At 18, a neurological examination revealed convergent strabismus in the right eye, a high palate, spinal scoliosis (Fig. 3), and contractures in the shoulders, wrists, iliolumbar joints, and pectoral muscles. Weak gluteal muscles and difficulty rising from an incomplete squat were also noted. Diffuse red patches of various sizes were present on the body’s skin, predominantly affecting the lower limbs and torso. Due to the rigidity of the paraspinal muscles, the patient cannot flex the thoracic and lumbar spine. Eosinophilia and neutropenia were consistently observed over 10 years (from age 10). A conducted WES study (at the age of 20) identified a homozygous variant c.100 + 1G > A in the *TBCE*.

### Clinical summary—P3

Patient 3 was previously briefly mentioned in the report discussing possible cases of sex development disorders in Ukrainian descendants (Globa et al. 2022). The patient is an 18-year-old man of Ukrainian descent with no significant family history. He was born at term with a birth weight of 2800 g (− 1 SD). Bilateral inguinal cryptorchidism was noted—orchidopexy was performed at the age of 3 and 4. Motor development was normal. At age 3, recurrent infections started, primarily acute respiratory viral infections, otitis, and bronchitis. The patient underwent a gonadectomy of the first testicle at the age of 10 and the second one at the age of 15; the histological report confirmed atrophic and sclerotic changes in the testicles and no signs of spermatogenesis. The patient was diagnosed with intellectual disability. A WES study (at 14) identified a homozygous variant c.100 + 1G > A in the *TBCE*.

A summary of the most pertinent clinical data is provided in Table 1, with comprehensive clinical information available in Supplementary Table 3.

**Table 1:** Clinical phenotype of reported *TBCE* c.100 + 1G > A homozygotes. *yo*, years old; *mo*, months old; *ENG*, electroneurography; *EMG*, electromyography

	P1	P2	P3
*TBCE* variant	c.100 + 1G > A (homozygote)	c.100 + 1G > A (homozygote)	c.100 + 1G > A (homozygote)
Age (current), sex	13, female	20, male	18, male
Genital abnormalities	None	Bilateral cryptorchidism	Bilateral cryptorchidism
Language development	Delayed (currently able to speak without dysarthria)	Delayed (currently able to speak without dysarthria)	Delayed (currently able to speak without dysarthria)
Movement development	Independent walking at 14 mo	Sitting up at 9 mo, independent walking at 15 mo (on tiptoes)	Normal
Thyroid and parathyroid function (TSH, FT3, FT4, PTH, calcium, phosphorus)	Normal	Normal	Normal
Dysmorphic traits	No	No	Yes
First symptom	Muscle weakness and atrophy (6 yo)	Febrile seizures, pneumonia (4 yo)	Acute respiratory viral infections (3 yo)
Repeated infections	No	Yes	Yes
Intellectual disability	No	No, ADHD diagnosis at 10	Yes
Eosinophilia	Yes, at least since 13 yo	Yes, at least since 10 yo	No
Neutropenia	Yes, at least since 13 yo	Yes, at least since 11 yo	Yes
Rigid spine	Yes, since 6 yo	Yes, since 10 yo	Not reported
Nocturnal hypoxemia	Yes, since 16 yo	Yes, since 16 yo	No data
EMG/ENG	At the age of 13. Normal muscle recording at rest and during exercise (the lower limit of normal). Normal nerve conduction	From the age of 10 to 20, EMG showed moderate myogenic features. Normal nerve conduction	No data
CNS MRI	At the age of 13. The corpus callosum thinned in the trunk area and was otherwise completely developed. No other significant deviations	At the age of 10—small hypophysis (5 × 8 × 13 mm). At the age of 20—no significant deviations	At the age of 16. Pituitary microadenoma suspicion. Epiphysis cyst. No other significant deviations
Muscle biopsy	No data	At the age of 11. Mild, non-specific myopathic changes	No data

The available clinical data of our patients, along with information obtained from the systematic review, have been compiled in Table 2 (Parvari et al. 2002; Hershkovitz et al. 2007; Sferra et al. 2016; Battini et al. 2021; Aminzadeh et al. 2020; Moussaid et al. 2012; Padidela et al. 2009; Albaramki et al. 2012; Ratbi et al. 2015; Kerkeni et al. 2015; Hafez et al. 2017; Naguib et al. 2009).

**Table 2:** Clinical phenotype of patients with reported *TBCE* pathogenic variants. IUGR, intrauterine growth restriction; *yo*, years old; *mo*, months old; *ENG*, electroneurography; *EMG*, electromyography

*TBCE* variants (No)	c.100 + 1G > A/c.100 + 1G > A (3)	c.464 T > A/c.464 T > A (5), c.464 T > A/c.1076del (1), c.464 T > A/c.924del (1)	c.155_166del/c.155_166del (91)
Auxologic parameters	Normal at birth (3/3, 100%)	Normal at birth (7/7, 100%)	IUGR (81/83, 97.6%)
Infantile development	Normal (3/3, 100%)	Normal (6/7, 85.7%)	Abnormal (47/47, 100%)
Age of symptoms manifestation	5.0 yo (SD 1.0)	6.3 mo (SD 4.0)	Neonatal period (48/48, 100%)
Signs at presentation	Muscle weakness and atrophy (1/3, 33.3%); Recurrent infections (2/3, 66.6%)	Hypotonia and developmental delay in the first year (5/7, 71.4%); spasticity and developmental delay in the first year (3/7, 42.9%)	Complications of hypocalcemia and hyperphosphatemia (44/48, 91.7%); growth delay (4/48, 8.3%)
Motor development outcome	Able to walk independently (3/3, 100%)	Able to sit (1/7, 14.2%); Never able to sit (1/7, 14.2%); Lost ability to sit (5/7, 71.4%)	No data
Speech and language outcome	Able to speak (3/3, 100%)	Able to speak with dysarthria (1/7, 14.2%); Able to speak with severe dysarthria (4/7, 57.1%); Not able to speak (2/7, 28.6%)	No data
Intellectual disability	1/3, 33.3%	7/7, 100%	36/37, 97.3%
Weight	Below 3rd centile (3/3, 100%)	Below 5th centile (1/3, 33.3%)	Below 3rd centile (29/29, 100%)
Height	Below 3rd centile (2/3, 66.6%)	Below 5th centile (1/3, 33.3%)	Below 3rd centile or “short stature” (90/90, 100%)
Thyroid and parathyroid function	Normal (3/3, 100%)	Normal (7/7, 100%)	Abnormal (69/69, 100%)
Microcephaly	0/3, 0%	1/7, 14.2%	40/40, 100%
Facial dysmorphism	1/3, 33.3%	0/7, 0%	69/69, 100%
Hypocalcemic seizures	0/3, 0%	0/7, 0%	41/43, 95.3%
Epilepsy not related to hypocalcemia	0/3, 0%	1/7, 14.2%	8/39, 20.5%
Neurologic findings	Rigid spine (2/3, 66.6%), proximal amyotrophy (2/2, 100%)	Distal amyotrophy, ataxia, spasticity—7/7, 100%	No data
Skeletal findings	Scoliosis (2/2, 100%)	Scoliosis (5/7, 71.4%)	Medullary stenosis (3/44, 6.8%)
Ophthalmologic findings	Strabismus (1/3, 33.3%)	Congenital cataract (0/7, 0%); optic atrophy (7/7, 100%); strabismus (3/7, 42.9%)	Congenital cataract (2/34, 5.9%)
Genital abnormalities in male patients	Cryptorchidism (2/2, 100%)	0/5, 0%	Cryptorchidism (3/23, 13.0%); small testes (2/21, 9.5%)
MRI CNS findings	No clinically significant changes (3/3, 100%)	Thin corpus callosum with moderate global cerebellar atrophy (4/7, 57.1%); additional white matter T2 hyperintensity in the posterior periventricular areas, T1 hypointensity areas in the pallidum and substantia nigra suggesting iron deposition and hypoplasia of the dorsal spine (2/7, 28.6%); Hypoplasia of the corpus callosum and mesencephalon, cerebellar atrophy, bilateral hyperintensity of peri dentate white matter, diffuse cortical and subcortical hypomyelinating-dysmyelinating leukodystrophy (1/7, 14.2%)	The thin corpus callosum, mild generalized lack of white matter, hypoplastic anterior pituitary gland with a thin infundibulum (6/6, 100%)
ENG	Normal (2/2, 100%)	Normal (6/7, 85.7%); axonal motor neuropathy (1/7, 14.2%)	No data
EMG	Normal (1/2, 50.0%); moderate myogenic features (1/2, 50.0%);	Normal (1/7, 14.2%); neurogenic features in distal and proximal muscles of legs (6/7, 85.7%)	No data

## Discussion

The clinical data obtained from patients harboring a novel pathogenic variant of the *TBCE* substantiate the existence of a broader spectrum of clinical symptoms than previously postulated. Our research encountered a notable challenge in the form of significant variations in the extent and precision of clinical descriptions across available reports (Parvari et al. 2002; Hershkovitz et al. 2007; Sferra et al. 2016; Battini et al. 2021; Aminzadeh et al. 2020; Moussaid et al. 2012; Padidela et al. 2009; Albaramki et al. 2012; Ratbi et al. 2015; Kerkeni et al. 2015; Hafez et al. 2017; Naguib et al. 2009). Nevertheless, the compiled data facilitate the derivation of several conclusions. The most severe clinical phenotype is unequivocally evident in patients with confirmed homozygous deletion c.155_166del, aligning with manifestations traditionally ascribed to HRDS or KCS1. Within this patient cohort, symptoms manifest prenatally, with intrauterine growth restriction identified in 81/83 cases (97.6%). Subsequently, most neonates (41/43, 95.3%) experienced seizure episodes linked to disturbances in calcium and phosphate serum levels. All patients displayed characteristic dysmorphic features, including a prominent forehead (5/6, 83.3%), low-set ears (38/38), depressed nasal bridge (6/6), thin upper lip (35/35), micrognathia (37/39, 94.9%), blue sclera (22/22), and microcephaly (40/40). Children’s development was marked by profound delays, with attained body mass and growth not surpassing the 3rd percentile. A high degree of intellectual disability (36/37, 97.3%) was consistently observed. Furthermore, it is noteworthy that patients presenting with the HRDS/KCS1 phenotype exhibit low survival rates in early childhood, with a majority succumbing before reaching the age of 2 (Hershkovitz et al. 2007).

In contrast to the above-mentioned group, patients with identified point mutations c.464 T > A presented milder phenotypes, different from those seen in the HRDS/KCS1, placing them in the spectrum of clinical manifestations. All children in this group exhibited normal auxologic parameters, and development in infancy for the majority fell within the normal range (6/7, 85.7%). Initial symptoms were observed on average at 6 months of age, primarily manifesting as hypotonia and developmental delay (5/7, 71.4%). Importantly, no endocrinological abnormalities in the form of hypocalcemia and hyperphosphatemia were observed. Nevertheless, the prognosis for these patients was poor, as none of the children achieved full verbal communication abilities—the majority exhibited severe dysarthria or a complete lack of speech development (6/7, 85.7%). All patients presented with intellectual disability, mainly in the moderate to severe range. Only one child achieved motor development, allowing independent sitting (1/7, 14.2%). Information regarding the height and weight of patients was available in three cases, where values below the 5th percentile were observed in only one patient. Interestingly, no dysmorphic features and genital abnormalities were observed. Another common finding in all children was the presence of optic nerve atrophy and abnormalities in CNS MRI examination. Neurogenic features in distal and proximal leg muscles were observed in 85.7% of patients, alongside normal motor and sensory nerve conduction recordings.

In the case of the homozygous point mutation c.100 + 1G > A carriers, the clinical course was most favorable and further distinguished by clinical symptoms not observed in previous groups. The patients were born at term during uneventful pregnancies. Bilateral cryptorchidism was observed in both male patients—a trait absent in all (5/5) male carriers of c.464 T > A variants. During infancy, none of the patients exhibited significant symptoms. Early motor development proceeded without notable delays. All patients have developed full speech abilities. Initial clinical manifestations occurred on average at the age of 5. No calcium and phosphate metabolism abnormalities or clinically significant changes in CNS MRI examinations were observed. Only in the case of P3 dysmorphic features has been observed alongside the intellectual disability. The absence of intellectual disability in patients P1 and P2 is important information, given that this trait is a clinical feature suggested in differentiating KCS1 from KCS2 (Schigt et al. 2023).

Patients P1 and P2 exhibited a prolonged course of eosinophilia and neutropenia. Patient P3 showed only discrete neutropenia. Interestingly, homozygous or compound heterozygous patients for the c.464 T > A *TBCE* variant did not exhibit recurrent infections (Sferra et al. 2016). In the case of patients P2 and P3, homozygosity for the c.100 + 1G > A variant was associated with recurrent infections as a prominent symptom during early childhood. Intriguingly, chronic neutropenia was identified in patient P1, who did not exhibit signs of immunodeficiency. Some reports noted that *TBCE* mutations impede polymorphonuclear cell chemotaxis and phagocytosis (Hershkovitz et al. 2007). This observation could explain why the total neutrophil count may not directly correlate with the manifestation of immunodeficiency symptoms. It should be noted that despite the frequent infections in the patient group with the HRDS phenotype, neutropenia was not reported in the literature. Only one report presented an 18-month-old female patient manifesting certain KCS1/KCS2 features with documented neutropenia and eosinophilia (Bergada et al. 1988). Unfortunately, this description predates the identification of the causative mutation. Hence, the diagnosis has not been confirmed through genetic testing.

Other manifestations occurring in P1 and P2 were nocturnal hypoxemia episodes. Sleep-disordered breathing was previously observed in 12 patients with HRDS phenotype (Al-Yaarubi et al. 2022). All patients were diagnosed with obstructive sleep apnea, while 4/12 had additional significant central apnea and sleep-related hypoventilation. However, this report does not explicitly document the confirmation of pathogenic *TBCE* variants within the patient cohort, and certain observed symptoms deviate from those typically seen in the HRDS. The etiology of sleep-disordered breathing in the HRDS and other genetically determined syndromes, such as Rett syndrome or Prader-Willi syndrome, has been postulated to involve abnormalities in craniofacial structure, obesity, decreased muscle tone, or structural abnormalities in the central nervous system (Al-Yaarubi et al. 2022; Tanizawa and Chin 2018; Gallego 2012). Still, none of the mentioned features were present in patients with the confirmed homozygous variant c.100 + 1G > A. Notably, this is one of the few *TBCE*-related symptoms that have not yet been observed in any case of KCS2 (Schigt et al. 2023).

In patients P1 and P2, an additional symptom was a rigid spine, a trait not previously documented in *TBCE*-related conditions. One report concerning monozygotic Chinese twins with KCS2 mentions severe spine rigidity in both individuals (Cheng et al. 2021). This symptom manifested around the age of 13, later than in the cases of patients P1 and P2 (6th and 10th year of life, respectively). Furthermore, in Chinese patients, a pronounced malformation of the cervical spine has been identified. It was characterized by a prominent odontoid process, with the tip extending above the anterior arch of C1, leading to compression with angulation of the spinal cord and myelopathy at the cervicomedullary junction level. Given the potential for serious complications associated with anatomical abnormalities in this region, we propose conducting imaging of the craniocervical junction for every KCS patient, including those with *TBCE* variants.

Distal amyotrophy, ataxia, and spasticity were observed in all patients with the c.464 T > A variant. In the case of patients with the c.100 + 1G > A variants, there were no signs of ataxia or cerebellar atrophy, but muscle atrophy and spasticity were present. However, in patients P1 and P2, muscle atrophy predominantly affected the proximal extremities and the shoulder and hip girdle muscles. Increased muscle tone primarily involved the paraspinal muscles causing a rigid spine. Importantly, in the case of P2, during numerous MRI examinations, features of atrophy and fatty infiltration of various muscle groups were confirmed (e.g., paraspinal muscles, iliopsoas, psoas, and buttock muscles). Muscle biopsy revealed discrete, non-specific myopathic changes. Description of muscle tissue in the MRI examination was available only for one other patient—the carrier of the c.464 T > A/c.924del variants (Battini et al. 2021). The examination revealed distal amyotrophy, particularly of the medial and posterior region of the thigh and calf, with the left side being more affected.

Although the last measured body weight of all c.100 + 1G > A carriers was below the 3rd percentile, unlike patients with other pathogenic *TBCE* variants, it was comparatively higher in the early years of life. In the case of P1, the decline in body weight below the 3rd percentile occurred at the age of 11, while in the case of P2, it occurred at 18. This was accompanied by a cessation of further height growth.

A common feature among patients with point mutations c.100 + 1G > A and c.464 T > A was the presence of skeletal system abnormalities, notably pronounced scoliosis (100% vs. 71.4%). In the case of patient P1, the T-score (− 3.9) at the age of 13 indicated the presence of osteoporosis, while in P2, the T-score (− 1.5) at the age of 14 suggested osteopenia. Such manifestation was not reported in c.464 T > A variant carriers (Sferra et al. 2016; Battini et al. 2021).

## Conclusion

Patients with the c.100 + 1G > A variant may manifest a distinct and broad phenotype. The primary clinical differences compared to homozygous c.464 T > A individuals include the onset of symptoms at a later developmental stage, normal motor development, eventual proper speech development, and cryptorchidism in boys. Additionally, CNS MRI studies revealed no significant pathology. Muscle atrophy primarily affects proximal muscles, and increased muscle tone is most pronounced in the paraspinal muscles, leading to spinal mobility limitations. Furthermore, patients exhibited recurrent infections, neutropenia, eosinophilia, and nocturnal hypoxemia. It should be acknowledged that pathogenic *TBCE* variants give rise to a broader spectrum of symptoms, and the analysis of this gene should not be confined solely to cases of patients presenting a phenotype classically described as HRDS/KCS1. Attention should be directed to significant differences in the prevalence of pathogenic variants concerning specific populations indicative of founder mutation effects.

## Supplementary Information

Below is the link to the electronic supplementary material.

Supplementary file1 (DOCX 21 KB)

Supplementary file2 (XLSX 64 KB)

Supplementary file3 (XLSX 20 KB)
