# Supplementary material for: Expanding TBCE-related phenotype—novel variant causing rigid spine, eosinophilia, neutropenia, and nocturnal hypoxemia
Source: J Appl Genet. 2024 Aug 17;66(2):363–73. doi: 10.1007/s13353-024-00894-9 (PMC12000180; doi:10.1007/s13353-024-00894-9)
Supplement: Supplementary file 1 — Supplementary file1 (DOCX 21 KB) [file 13353_2024_894_MOESM1_ESM.docx]

| **Patient 1 -** Whole Exome Sequencing |
| --- |
| Laboratory: CeGaT GmbH (Germany).  The raw sequencing data was converted to FASTQ format using Illumina's software (bcl2fastq) and aligned to the human reference genome (GRCh37/hg19) with Burrows-Wheeler Aligner (BWA-MEM). Following alignment, GATK algorithms (Sentieon) were employed for duplicate read marking, local realignment around indels, base quality score recalibration, and variant calling for nuclear DNA (nDNA). Annotation of variant data utilized various tools (VcfAnno and VEP) and incorporated public variant databases such as gnomAD, ClinVar, and HGMD. Median sequencing depth and coverage across target regions were determined based on MQ0-aligned reads. Quality control measures included in-process reference samples to ensure sensitivity and specificity thresholds were met, with thorough assessments for contamination and sample mix-up performed on the patient's sample. Copy number variations (CNVs), encompassing single exon or larger deletions or duplications (Del/Dups), were identified using a proprietary bioinformatics pipeline. Differences between observed and expected sequencing depth were calculated to delineate regions with variable DNA copy numbers. The expected sequencing depth was derived from other samples processed in the same sequence analysis. Additionally, adjustments were made for guanine and cytosine content variations in the sequence data. The potential pathogenicity of identified variants was evaluated by considering factors such as predicted consequence, biochemical properties of codon changes, degree of evolutionary conservation, and data from reference population and mutation databases including, but not limited to, the 1000 Genomes Project, gnomAD (<https://gnomad.broadinstitute.org/>), ClinVar (<https://www.ncbi.nlm.nih.gov/clinvar/>), and HGMD Professional (<http://www.biobaseinternational.com/product/hgmd/>). Missense variants underwent further classification using in silico prediction tools like SIFT, PolyPhen, and MutationTaster. Additionally, the clinical relevance of any detected copy number variations (CNVs) was determined through a literature review and consultation with databases such as the 1000 Genomes Project, Database of Genomic Variants (http://dgv.tcag.ca/dgv/app/home), ExAC, gnomAD, and DECIPHER (https://www.deciphergenomics.org). For mitochondrial DNA (mtDNA) variants, specific databases like Mitomap, HmtVar, and 1000G were referenced. The clinical evaluation team assessed variant pathogenicity based on patient referral information, literature review, and manual inspection of sequencing data when necessary. Reporting adhered to HGNC-approved gene nomenclature and mutation nomenclature following HGVS guidelines. Sequence variants categorized as pathogenic, likely pathogenic, and variants of uncertain significance (VUS) underwent confirmation via bi-directional Sanger sequencing if they did not meet stringent NGS quality metrics to ensure a true positive call. |
| **Patient 2 -** Whole Exome Sequencing |
| Laboratory: BluePrint Genetics (Finland).  Protein-coding regions, flanking intronic regions, and additional disease-relevant non-coding regions were enriched using in-solution hybridization technology and sequenced using the Illumina NovaSeq 6000/NovaSeq X Plus system. Illumina bcl2fastq2 was used to demultiplex sequencing reads. Adapter removal was performed with the Skewer. The trimmed reads were mapped to the human reference genome (hg19) using the Burrows-Wheeler Aligner. Reads mapping to more than one location with identical mapping scores were discarded. Read duplicates that likely result from PCR amplification were removed. The remaining high-quality sequences were used to determine sequence variants (single nucleotide changes and small insertions/deletions). The variants were annotated based on several internal as well as external databases. Variants were classified and reported based on ACMG/ACGS-2020v4.01 guidelines (Richards et al., 2015, PMID: 25741868,<https://www.acgs.uk.com/quality/best-practiceguidelines/>). Only variants (SNVs/Small Indels) in the coding region and the flanking intronic regions (±8 bp) with a minor allele frequency (MAF) < 1.5% were evaluated. Known disease-causing variants (according to HGMD) were evaluated in up to ±30 bp of flanking regions and up to 5% MAF. Minor allele frequencies were taken from public databases (e.g., gnomAD) and an in-house database. If high-throughput sequencing did not achieve an acceptable sequencing depth per base, local re-sequencing using classical Sanger technology was performed. |
| **Patient 3 -** Whole Exome Sequencing |
| Laboratory: Human Developmental Genetics, Institute Pasteur (France).  Whole exome sequencing (WES) was conducted using Agilent SureSelect Human All Exon V4 for target enrichment, followed by paired-end sequencing on the Illumina HiSeq2000 platform employing TruSeq v3 chemistry. Subsequent data analysis utilized the manufacturer's proprietary software specific to the sequencing platform. All reads were aligned against the human reference genome (NCBI, GRCh37/hg19, or GRCh38/hg38) through the Burrows-Wheeler Aligner. Single-nucleotide variants, small insertions, and deletions (InDel) were selected with GATK (v1.6). Picard (v1.62) (<http://broadinstitute.github.io/picard/>) and SAMtools (v0.1.18) were utilized to mark duplicate reads and to process the BAM file manipulations. Single-nucleotide polymorphism (SNP) and indel variants were annotated to dbSNP 138 identifiers using the Genome Analysis Toolkit (GATK) Unified Genotyper. The SNP Effect Predictor bioinformatics tools from the Ensembl website (<http://www.ensembl.org/homosapiens/userdata/uploadvariations>), gnomAD website (<https://gnomad.broadinstitute.org/>), and ClinVar website (<https://www.ncbi.nlm.nih.gov/clinvar/>) were aplied to annotate the novel variants, followed by manual screening of all variants in the Human Gene Mutation Database Professional Biobase (<http://www.biobaseinternational.com/product/hgmd/>). Sanger sequencing was performed to confirm potentially pathogenic variants. |

**Supplementary Table 1.** Methodology overview for Whole Exome Sequencing in Patients 1-3.
